# Social Deprivation and Cognition in Older Adulthood: The Mediating Role of Lifestyle Activities

**DOI:** 10.1002/alz.085539

**Published:** 2025-01-09

**Authors:** Lena M Hofbauer, Francisca S Rodriguez

**Affiliations:** ^1^ German Center for Neurodegenerative Diseases (DZNE), site Rostock/Greifswald, Greifswald Germany; ^2^ German Center for Neurodegenerative Diseases (DZNE), site Rostock/Greifswald, Greifswald, Mecklenburg‐Vorpommern Germany

## Abstract

**Background:**

Previous research has shown that the experience of social deprivation is associated with impaired cognition in older adulthood. It has been proposed that this may be explained by social deprivation being associated with a less ‘brain‐healthy’ lifestyle. We thus investigate mediating effects of lifestyle in the association between social deprivation and cognition.

**Methods:**

Data of 3,015 respondents (Mean Age: 74.68 years, SD: 6.49; Mean Follow‐Up 6.40 years, SD: 2.16) in the Health and Retirement Study (HRS) were included in the analysis. Social Deprivation Status was evaluated using scores on the Social Deprivation Index. Further, we constructed a Lifestyle Index to measure engagement in lifestyle activities. The final index reflects the frequency at with respondents attend educational/training courses, read, do writing, go to non‐religious organisations or clubs, volunteer, and spend time on a hobby/project. Growth curve modelling was used to model cognition over time. Path modelling was used to evaluate the mediation.

**Results:**

While a higher Social Deprivation Status was associated with poorer cognition, a higher Lifestyle Index score was associated with better cognition (β = 1.56, [95% CI: 1.23, 1.89], *p<.001*). The association with change over time was significant but negligible (β = 0.00, [95% CI: 0.00, 0.00], *p = .026*). Mediation analysis showed that the Lifestyle Index score accounted for 14.8% and 23.5% of the effect of Social Deprivation Status on cognition at baseline and last follow up, respectively.

**Conclusions:**

Lifestyle is a partial mediator in the association between social deprivation and cognition. Yet, the size of the mediation indicates that its contribution is limited and broader factors, such as structural disadvantage, are likely at play. Thus, prevention efforts should not be limited to individual lifestyle but rather consider broader factors associated with social deprivation.

